# Arc Tracking Control in Insulation Systems for Aeronautic Applications: Challenges, Opportunities, and Research Needs

**DOI:** 10.3390/s20061654

**Published:** 2020-03-16

**Authors:** Jordi-Roger Riba, Álvaro Gómez-Pau, Manuel Moreno-Eguilaz, Santiago Bogarra

**Affiliations:** 1 Electrical Engineering Department, Universitat Politècnica de Catalunya, 08222 Terrassa, Spain; bogarra@ee.upc.edu; 2 Electronics Engineering Department, Universitat Politècnica de Catalunya, 08222 Terrassa, Spain; alvaro.gomez-pau@upc.edu (Á.G.-P.); manuel.moreno.eguilaz@upc.edu (M.M.-E.)

**Keywords:** Arc tracking, partial discharges, corona effect, insulator materials, polymers, power system protections, aeronautic wires

## Abstract

Next generation aircrafts will use more electrical power to reduce weight, fuel consumption, system complexity and greenhouse gas emissions. However, new failure modes and challenges arise related to the required voltage increase and consequent rise of electrical stress on wiring insulation materials, thus increasing the risk of electrical arc appearance. This work performs a critical and comprehensive review concerning arc tracking effects in wiring insulation systems, underlying mechanisms, role of materials and possible mitigation strategies, with a special focus on aircraft applications. To this end an evaluation of the scientific and technological state of the art is carried out from the analysis of theses, research articles, technical reports, international standards and white papers. This review paper also reports the limitations of existing insulation materials, standard test methods and mitigation approaches, while identifying the research needs to comply with the future demands of the aircraft industry.

## 1. Introduction

The next generation of more electric aircrafts (MEAs) is being developed with the final goal of evolving towards all-electric aircrafts (AEAs), since they are environmentally friendlier [1]. To this end, it is required to optimize the performance, reliability and power density, by lowering the weight [2], reducing Operations and Maintenance (O&M) costs, reducing system complexity, fuel consumption and greenhouse gas emissions of the new aircrafts. MEAs and AEAs will use more electrical power than their predecessors, since they replace pneumatic and hydraulic systems by electrical ones [3] to reduce the weight. Therefore, they are required to operate at higher voltage levels [4], above 1 kV [5], higher dV/dt, higher power densities and decreased wire distances. These modifications lead to new types of failure modes, including a higher risk of electric arc appearance, which can be potentially harmful [6]. A foremost concern related to electric arc formation in aeronautic environments is the presence of foreign object debris, since a conductor object can induce an electric arc when shorting out two wires [6]. Electrical systems well-designed for ground operations can generate partial discharges (PDs) when operating at lower pressures; this effect is frequently intensified due to moisture condensation caused by pressure changes during rapid ascents and descents [1]. Therefore, aeronautic and aerospace insulation systems are more susceptible to PDs because of the low pressure operating environment [7], with inception voltages lower than those found at sea level [2], especially when operating at higher voltage levels [8,9].

Since electric and electronic devices are being increasingly applied in aeronautics and aerospace sectors, polymer insulation materials are inexorably exposed to a wide range of environments. However, such demanding applications require materials and devices with very reliable operation under a wide range of environmental and operating conditions [10].

PDs are electrical discharges that do not completely bridge the insulation between two electrodes [11]. They are ignited by three fault types, i.e., internal, external and corona. Persistent PDs can produce severe insulation damage due to the chemical transformations produced by the flow of electrons in the defect. Such transformations can produce a conductive path, which allows the flow of an electric current and a rise of the temperature, thus damaging the insulation and promoting the formation of an arc or complete breakdown due to the weakening of the insulation [12]. Arcing can be generated between electrical wires separated by a solid insulating medium when the later one becomes carbonized. When the carbonization is generated because of an electric current, the arcing process is known as arc tracking. 

**Figure 1:**
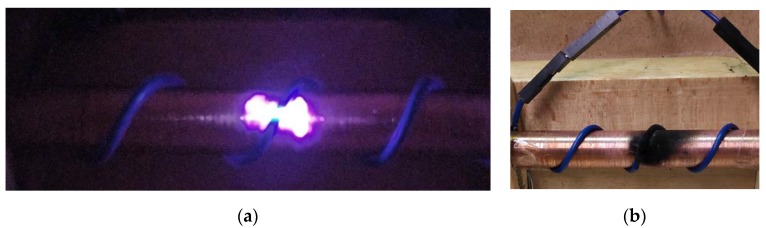
(**a**) Initial corona discharge at 10 kPa under 600 V–50 Hz. (**b**) Effect of arc tracking in a conductor.

Electrical tracking is originated by surface PD activity on an organic material, which degrades the polymer, thus moving from the insulator to conductor states due to powerful thermal shocks produced by an intense bombardment of electrons [13], which contribute to break the polymeric chains and, ultimately, to generate carbonized conducting paths. New discharges extend existing tracking paths, thus reducing the region of healthy insulation. The applied voltage will generate other carbonized paths on the healthy insulation surface between the high voltage electrode and ground, thus leading to a flashover or a breakdown [14], with the consequent fire hazard [15]. Therefore, arc tracking can be understood by self-sustained arcing between two or more wires across a carbonized path produced due to degradation of the insulation, which can induce ignition of wiring [16], even in 12 volt automotive wiring systems [17] or explosions [3]. Figure 1 shows an initial corona discharge and the effect of arc tracking in a conductor.

There is a lack of technical literature focused on the analysis of arc tracking, and more specifically centered on aeronautic applications. This shortage of data is due to the difficulty of arc characterization because of its very unpredictable behavior [18].

This paper reviews the state of the art related to arc tracking, with a special emphasis on insulation wiring systems for aircraft electrical and electronic systems, focusing on the current trends in MEAs and AEAs. Due to the increased proportion of electrical power in next generation aircrafts, insulation materials have to withstand increasing levels of electrical stress, thus being more prone to electrical discharge activity. The paper reviews the current understanding related to the mechanisms leading to arc tracking, as well as the effects and possible remedial actions to neutralize or minimize arc tracking occurrence. This review paper aims at performing a comprehensive and critical analysis of such effects under aeronautic environments, while detecting the research needs and challenges to be faced. This work is based on information and data found in international standards, recent scientific and technical publications, including journals, conference papers, theses, white papers and technical reports.

The rest of the article is organized as follows. Section 2 is devoted to presenting the factors affecting arc tracking with special emphasis in aeronautic wire insulation materials. A profuse review of the state of the art is provided, disclosing the conditions and circumstances of arc tracking in aeronautic wires. Section 3 focuses on the two major situations of aeronautic wire arc tracking, namely, wet-wire and dry-wire arc tracking. Section 4 exposes the main types of wires used in the aircraft industry, disclosing their pros and cons as well as highlighting the ups and downs suffered over the years in the search of new and better materials. Section 5 is devoted to the discussion of the existing standards for assessing the arc tracking prevention capabilities of commercial aeronautic cables. Section 6 explains the main arc tracking sensing, detection and processing mechanisms found in the related literature. Their principles of operation are uncovered, as well as the adopted aeronautic industry implementations are discussed. Section 7 lists the identified research needs in regard to the studied field, aiming to serve as future research guidelines that will meet the challenges disclosed throughout this review article. Finally, Section 8 summarizes the main ideas and concludes the paper.

## 2. Factors Affecting Arc Tracking

Polymeric materials with low weight and standing electrical and mechanical properties are widely utilized in electric insulation applications [15,19,20]. However, most of such organic materials can be damaged by tracking due to the change in the carbon bonds in their inner structure, and thus, the polymer resistance towards electrical discharges is much lower compared to inorganic insulator materials such as glass or porcelain [21]. Research on tracking resistance of insulation materials is a key point to ensure the long-term reliability [15] and safety of electrical insulation systems [22]. Recent studies show that polymeric nanocomposites tend to offer considerably enhanced properties compared to conventional and micron-sized particle-filled polymers [23,24]. It is proved that with the addition of nano- and micro-fillers hydrophobicity increases, whereas wettability, surface energy and interaction parameters tend to decrease [23]. In [24] it is shown that the resistance to tracking and erosion, the resistance to high-voltage arcing and the resistance to thermal degradation can be improved substantially with low nano-filler contents. Such improvements, which appear for filler contents usually below 10 wt.% [24], arise from the strong interfacial interactions and ultra-fine phase dimensions, thus improving hydrophobicity, erosion and tracking resistance [23], as well as resistance to PDs and treeing performance [24]. The interphase plays a key role, since it can present different properties from those of the uninfluenced polymer [24]. 

Insulation materials are subjected to mechanical, electrical and environmental stresses [25], as well as electrostatic discharges, which may generate cracks in wiring insulation materials [26]. In particular, environmental stresses such as ultraviolet radiation from sunlight, moisture, contaminants and pollution such as ozone or acid rain [27] and aged polymeric insulation materials [28] could degrade the material due to surface tracking [29] and erosion [30]. Wire insulation materials present different unanticipated failure modes, such as arc tracking, arcing and finally insulation flashover in order of increasing severity [31]. 

An electric arc is defined as a luminous high temperature electric discharge through a medium such as charred insulation or across a gap [32] under AC or DC supply [33]. Inner arc temperatures are in the range of several thousands of Kelvin degrees, although they depend on several factors, including voltage drop, current, geometry or materials involved [34]. Usually, radiative losses lessen as pressure decreases, and thus arc temperature increases [4]. Arc behavior, ignition and extinction depend on the conditions of the electrical network, voltage level and type (AC, DC, shape of the waveform, polarity and different frequencies), as well as on other factors such as environmental conditions, specifically pressure, water condensation, gases, conductor material, thickness and composition of the insulation. In [35] it is stated that DC arc characteristics are also influenced by the chemical nature of the wetted layer at the surface of the solid insulator. Other important factors are wire design and aging parameters. To sustain the arc, minimum current and voltage conditions are required. If those threshold values are not reached, the arc will not be ignited [16]. In [36] an attempt to model the behavior of DC arcs is made under aeronautic pressure conditions, although different assumptions must be carried out to simplify the inherent complexity of the arc nature, including that the arc is supposed a one-dimensional radial problem, the arc has cylindrical shape, the arc column is at thermodynamic equilibrium and the electric field within the arc column is uniform, convection is omitted so only power losses due to radial thermal conduction are taken into account, and the arc power in the electrode surface can be expressed as the product of the electrical current by the equivalent voltage due to the arc. According to this simplified one-dimensional model, the arc voltage *V_arc_* versus the length of the arc column length can be expressed as,

$$V_{arc}=V_{A}+V_{C}+\int_{0}^{d}E\left(x\right)dx,$$

where *V_A_* is the voltage drop in the anode, *V_C_* is the voltage drop in the cathode, *E* is the mean electric field strength in the arc column and *d* is the length of the arc.

In [37] a non-adiabatic Finite Element Method (FEM) model of the arc is proposed, which can be applied to model dry and wet arc tracking effects.

In AC circuits, the arc extinguishes at each current zero. Under vacuum conditions, the arc lasts less, stopping fast after the first current zero, although the effects of the arc are more severe compared to ambient pressure [38]. Under DC conditions, a minimum current is required to stabilize the arc, otherwise it tends to self-extinguish. When the minimum threshold current is applied, the arc sustains providing that the current is not switched off by the protections. Since the rated current to activate the protections usually depends, primarily, on the diameter of the wire, greater damage is expected for larger diameters [16].

Experimental results for DC tracking conducted under different pressure levels conclude that the relationship between tracking failure and pressure and the type of damage depends on the type of insulation material. Some materials undergo tracking-type damage, whereas others undergo erosion-type damage. Erosion occurs in polymer materials that do not generate carbonized tracks, the surface being eroded progressively due to the gaseous discharge, finally leading to dielectric breakdown [22]. It was reported that the AC tracking resistance is higher than the DC one, and that the DC tracking resistance decreases when decreasing the pressure [22].

Tracking typically arises due to surface contamination [39]. Arc tracking usually requires a polymeric insulating surface contaminated with humidity, liquids, conductive dusts or salts [19,32], placed in a circuit with a sufficient voltage applied, contamination generating a closed electrical circuit across the polymer surface, and time. Arc tracking corresponds to the propagation of the arc along the wires due to ablation of the metallic core and degradation of the insulation [4]. The discharge path can be produced due to the existence of debris or conductive materials on the surface or even due to the conductivity of the insulation itself. When the discharge develops with a sufficient length, it can evolve to flashover. These short duration short-circuit arcs between a faulty polymeric insulated wire and another wire [40] can partially pyrolize (thermal carbonization) the insulation [41], thus generating an electrical conductance along the surface of the insulation. Small leakage currents flowing across the contaminated area degrade the base material, producing arc discharge, charring or igniting combustible substances nearby the arc. This problem, typical of high-voltage systems, has also been described in 120/240 V AC systems [32]. The charred polymer becomes conductive, being able to sustain a short-circuit arc, which may spread along the wire due to a continuous pyrolyzation of the insulation material, leading to the arc tracking effect [41] due to the formation of a conductive carbon path along cracks within the insulation. In the case of multiple wire bundles in a harness, the insulation layers of other wires within the bundle may also become thermally carbonized or charred, thus starting to arc track, and ultimately leading to a complete fault of the entire bundle or even the harness [42], producing a massive current leakage and leading to the flash-over [43] and ultimately resulting in severe functionality loss [44]. Arc damage in a wire bundle develops in a hybrid arrangement including insulation materials, metallic conductors, and arc plasma. Depending on electrical network parameters including the power source and the equivalent impedance, such faults may lead to stabilized arcs as long as the protections are not activated and a self-extinguishing process is initiated. Once the arc has been extinguished and the system reenergized, the phenomenon can reemerge, depending on applied voltage, load current, chemical composition and thickness of the partially damaged insulation [45].

Because of the electric resistance of the carbon path, the temperature along the conductive path increases due to the localized heat generated by the flow of a leakage current, thus pyrolyzing the insulation. Despite the small size of arcing, it can ignite combustible gases produced on the surface [41]. Arc tracking occurs because the polymer surface becomes conductive, heating up the polymeric material and finally causing ignition [46]. Therefore, arc tracking transforms an insulating material from non-conductive to conductive through a surface degradation process [46]. In [47], thermal spots with temperatures above 400 °C are reported. This heat ultimately leads to a chemical breakdown of the insulation area surrounding the carbon path, generating more carbon along the path, thus self-propagating the phenomenon and tending to grow with time. Arc tracking duration may fluctuate between some tenths of second and few seconds, generating current and voltage pulses in a wide spectral range [48]. In [49] it is proved that during the inclined plane test, dry band discharges (DBDs) have different stages. First, DBDs in the form of streamer discharges and partial arcs tend to stay at the same position until the contaminant is completely evaporated. Next, DBDs occur faster and change their location due to the reduction of the sample resistance because of the generated carbonization. Finally, the discharges are longer, emit more light and the leakage current increases compared to the previous stages.

Proneness to tracking is heavily influenced by the chemical composition of the insulation [16]. It has been found that some insulation materials can be inadequate for use in high altitude applications [22]. Apart from the effects of the contaminants, arc tracking can also be triggered by different factors, including radial cracking, imprint labeling, abrasion by rubbing between wires or between a wire and the airframe [32] or by short circuits. Arc tracking can also be accelerated due to undetected damage in inaccessible areas or also by using unsuitable insulation types in areas prone to severe weather and moisture conditions [43]. The minimum current required to initiate arc tracking depends on the specific insulation, since insulation materials have different arc tracking propensity, types and test conditions. Materials prone to carbonization, i.e., aromatic polymers, thermoset plastics, or materials including alternating double bonds, are more susceptible to arc tracking, although there are no plastics or polymers completely resistant to arc tracking [17].

It is known that surface treatments may increase the arc tracking resistance of polymeric materials. In [25] it is proved that by fluorinating silicon rubber (SiR) samples by means of an F_2_/N_2_ mixture, flashover tests show an improvement of the tracking resistance. SiR insulation systems are additivated with different filler materials that are used to increase the hydrophobicity. Resistance to UV radiation can also influence tracking and erosion behavior [50]. The traditional solution to prevent arc tracking consists in the use of copper wires with an insulation composed of polytetrafluoroethylene (PTFE) fluorocarbon polymer [18].

Studies performed on insulations combining cross-linked polyethylene (XLPE) and SiR indicate that increased values of the interfacial pressure can delay interfacial discharges and tracking failure, since it confines the initial interface discharges and restricts the expansion of carbonization paths [51]. However, when increasing the interface pressure, the size of the discharge channel reduces, and the damage inside the samples increases [52]. Such studies also indicate that the minimum voltage to initiate surface discharges increases with the ambient pressure, which also impacts the distribution characteristics of the light emitted by the discharges and the tracking pattern at the interface [53]. 

## 3. Wet- and Dry-Wire Arc Tracking

Polymers require a continuous and large thermal energy supply to decompose, i.e., to evolve volatile molecules [54]. High temperature discharges in polyimide (PI) materials tend to produce thermal degradation due to the carbonization of the aromatic polyimide. Initially, carbonization takes place at localized spots but as the reaction continues, a carbon path can be formed between conductors, so the heat produced by the high electric current flowing through the carbon path will cause severe arcing, eventually leading to flashover [55]. It was shown that by means of thin fluoropolymer coatings, certain polyimide insulation (Kapton®) can be improved to endure wet arc tracking [55]. When ageing, Kapton® insulation becomes mechanically more vulnerable and brittle, thus generating cracks in the insulation and favoring arc tracking appearance. Therefore, the tendency is to discontinue Kapton® wires in new aircrafts [56]. Once the Kapton® layer becomes cracked, the wiring is susceptible to three potential issues, i.e., hydrolysis (insulation cracking and breakdown due to exposure to moisture), wet arc tracking, and dry arc tracking [44]. Most aliphatic fluoropolymers used in wire insulation, except cross-link-Ethylene tetrafluoroethylene (XL-ETFE), do not generate carbon deposits due to thermal degradation, and some polyimide-fluoropolymer insulation materials can resist wet arc tracking [55]. Both wet and dry arc tracking, when occurring in wires with aromatic polyimide insulation (Kapton®), can lead to carbon arc tracking. Wet and dry arc tracking can produce very harmful effects in insulation systems and lead ultimately to a real hazard for the whole aircraft [44]. It is known that PVC-insulated wires are prone to fail due to self-induced wet-tracking, especially when placed in very low humidity atmospheres [57]. 

Wet-wire arc tracking (WWAT) is a specific type of arc tracking that occurs when leakage currents originated on a wet insulating surface are able to vaporize its moisture, forming dry spots, which offer a high resistance path to the flow of the electric current. The leakage current can generate surface discharges, resulting in the degradation of the material [58]. As a result of the leakage current, a voltage will be induced across the spots due to the leakage current, thus developing tiny surface discharges, firstly appearing as surface scintillations. Scintillations are arcing surficial electrical discharges, being low-energy and low-temperature events. But if the process continues unrestrictedly, it may lead to full-developed arcing in the air gap between two wires, ultimately generating high temperatures and being quite destructive [17]. However, scintillations can generate spot temperatures of about 1300 K, thus thermally degrading the insulation to a certain extent, depending on the nature of the insulation material [55]. Therefore, wet arc tracking can occur when moisture or aircraft fluids induce a short circuit between two adjacent exposed wires or between an exposed wire and the aircraft structure, which are at a different electric potential. Conversely, dry arc tracking occurs mainly due to insulation abrasion or damage or bad installation practices under dry conditions, therefore generating a short circuit between two adjacent wires [44]. Dry arcing generated by nearby carbonized areas promotes thermal degradation, thus producing tracking and erosion [47,59].

Hydrophobicity tends to delay leakage current development [59]. However, due to the presence of moisture and contamination, the outer layer of the insulation can change from hydrophobic to hydrophilic [25]. Since this wet layer is conductive, a leakage current is generated in this new conductive layer. When the wire dries, this conductive layer tends to break, and dry bands are formed with a consequent voltage drop across each dry band. If the voltage applied is high enough, the air surrounding the surface will break down, thus forming dry band arcs [60]. 

In [18] it is proved that 50%–65% of the power associated with arc tracking between adjacent wires is transferred to the wires, thus melting and partially vaporizing the metallic core and insulation, being radiated or conducted. The remaining energy is transferred to the arc column, being conducted, convected or radiated.

It was shown that low-energy corona discharges and UV radiation can break C–H and Si–CH3 bonds of SiR insulation, thus converting to hydrophilic the initially hydrophobic polymer surface [25]. Once contaminated or oxidized, SiR cannot easily recover hydrophobicity. This effect increases the leakage current on the contaminated surface under wet conditions, facilitating dry band arcing, which is a highly energetic phenomenon and can seriously damage the surface by tracking and erosion, thus reducing the mechanical strength and insulating properties significantly [47].

Both wet and dry arc tracking can lead to very harmful effects in insulation systems and ultimately to a real hazard for the whole aircraft.

## 4. Wire Types for Aircraft Applications

Aircraft wiring is a key element in transmitting electrical power and signals along the aircraft. With the development of new MEA systems, aircraft wires are becoming increasingly demanding in terms of light-weight, long-life requirements and electrical and physical characteristics [61]. Aircraft wiring is often subjected to diverse factors such as temperature and pressure changes, vibration, moisture, or chafing. Whereas many aircrafts parts are periodically inspected or renewed, due to its complexity, the wiring system remains usually untouched, thus favoring the conditions for the occurrence of electrical arc faults [62]. 

Insulation wire damage can induce a high-current arc between two metallic conductors, which can cause an electrical breakdown of the whole system [38]. Therefore, fault arcs generated in aeronautic circuits must be eradicated or controlled to limit their effects [4]. Today, wiring systems are a focus of consideration due to diverse aircraft accidents. According to the FAA [31], wire insulation materials are selected by balancing mechanical, electrical, thermal and chemical properties, whereas low smoke and non-flammability are a plus. Wire insulation materials are chosen based on the type of installation, so their features are selected depending on the operating environment, including arc resistance, dielectric strength, corrosion and abrasion resistance, fluid resistance, mechanical strength, impact strength, cut-through strength smoke emission, heat distortion temperature, or flame resistance, among others [63,64,65,66]. Heat-aging behavior must be considered when selecting electric wires, since they are often placed in areas under extreme operating conditions (current loading, temperature and pressure ranges) with infrequent inspection over long periods of time. Thus, heat resistance is a key factor in the selection and rating of aircraft wire [63].

At the end of the sixties, tetrafluoroethylene (TFE) and PTFE (Teflon®) were the most used aircraft wire insulation materials because of the excellent physical, electrical, mechanical and chemical properties, although limited to operating at 200 °C. ETFE (Tezfel®) appeared during the seventies as wire insulation material since it allowed a great reduction of the wire weight and diameter while exhibiting good physical, electrical, and corrosion properties, although it is a soft material, thus making its installation more difficult. However, ETFE use was limited to operating below 150 °C. During the late seventies, the radiation crosslink method was successfully adapted to ETFE insulation, so XL-ETFE became widely applied due to an operating temperature of 200 °C, and better mechanical properties than PTFE [67]. Today, XL-ETFE is still broadly applied. PI wire insulation tape was developed during the early seventies [68]. PI film insulation exhibits appealing features such as low weight, high dielectric strength, non-flammability, and high temperature capability [69]. PI is a self-extinguishing polymer, has a decomposition temperature beyond 500 °C, and can operate at 250 °C~280 °C, while offering excellent physical, electrical, and mechanical properties and generating low smoke density when burning. However, several accidents due to its tendency to hydrolyze, resulting in low moisture and arc resistance, generated awareness of its widespread use. Upon arc initiation, degraded PI insulation becomes conductive, and the arcs can propagate along the wiring harness [69]. Therefore, some aircraft manufactures banned its use. During the nineties, aircraft wire manufacturers combined PTFE with PI to produce PTFE/PI tape covered with PTFE tape, allowing a very thin insulation layer, thus saving weight compared to other wires. PTFE/PI tape wrapped wire has an operating temperature of 260 °C [61]. Insulation materials with a high PI ratio exhibit a shorter arc duration with respect to wires with ETFE and PTFE insulation, the last ones being characterized by a high arc resistance due to the self-extinguishing features of the arc and the longer time to breakdown. Therefore, wires with ETFE and PTFE insulation are more stable against a fault arc than those with PI insulation [38].

Currently, the two most applied wires in commercial aircrafts are insulated with Kapton® (aromatic polyimide) or with a fluoropolymer (TFE, FEP, ETFE, or other fluoropolymer resins) [55]. Although used in the past and still installed in old aircrafts, insulation materials such as PVC/nylon, Kapton® or Teflon® are almost no longer installed in new aircrafts. New aircraft designs mainly use insulation materials made of Tefzel®, TKT (Teflon®/Kapton®/Teflon®) and PTFE/Polyimide/PTFE [63]. Table 1 summarizes the typical wiring used in commercial aircrafts, according to the FAA [54].

The AS50881 aerospace standard [70] guides how to size both the conductor and insulation intended for aerospace wiring. Conductor size depends upon the current carrying capability, taking into account the use of conductor bundles and the operating altitude, whereas insulation thickness relies on the rated voltage, so that it must withstand PD occurrence [71].

## 5. Comparative Tracking Index (CTI)

The CTI is applied for measuring the arc tracking properties of an insulation material. The test method is detailed in the IEC standard 60112 [72], which defines the CTI as the maximum measured voltage expressed in volts, at which a material withstands 50 drops of polluted water without generating tracking and a persistent flame. The ASTM international standards organization has an equivalent standard, the ASTM D368 [73].

The international standards ASTM D2303 [74] and IEC 60587 [39] are widely applied for ranking the resistance to erosion (loss of material due to electrical discharge or leakage current) and tracking in insulation materials, by means of a liquid contaminant and an inclined plane [75]. The inclined plane standard test method [39,74] is commonly used to measure erosion and tracking resistance of insulation materials during the design process but not for monitoring purposes, since it emulates the thermal degradation by electric arcing. However, it is a complex and time-consuming method, thus very difficult to perform in field inspection [76] and it has an inherent limitation since only a 4-mm gap can be applied, due to the polluted liquids dropping system defined in the standard [71].

The inclined plane test presents other issues. It was reported that the effects of DC dry arcing on polymeric materials are severer than those due to AC dry arcing, although it was attributed mostly to factors including electrohydrodynamic effects, test electrodes corrosion, or deposits formed by liquid contaminants. Therefore, it is required to validate the suitability of the methods and apparatus specified in the standard AC inclined plane test (the most widely applied screening method) to conduct DC tests, and to conclude whether the greater harshness of such DC dry arcing effects, relative to AC dry arcing, is due to the apparatus and methods applied or due to fundamental underlying mechanisms [75]. Therefore, there is a need to develop an equivalent standard test method for DC.

## 6. Arc Tracking Detection Methods

Today, modern aircrafts use arc fault circuit breakers (AFCBs) as protection against arcing effects. AFCBs are based on electronic components. Due to electrical arcing event occurrence, by analyzing the current waveform, the circuit breaker trips within 100 ms, according to the AS6019 standard [77]. When the fault occurs, the waveform is altered, and the AFCB must identify the arc fault and disconnect the circuit affected by the fault. However, AFCBs act once the arcing occurs but not before, so they can be improved to reduce the damage level and shorten the reaction time. With the next generation of MEAs, which will depend more and more on electrical and electronic systems, the use of current AFCBs is questionable [62], so it is required to develop new approaches for an early detection.

Early symptoms of arc tracking include partial discharges (PDs) and corona. PDs are electric discharges which only partially bridge the insulation material between two electrodes [11]. PDs deteriorate the insulation due to the bombardment of discharge ions and the action of chemicals formed during the discharge. In most MEA systems, the discharges occur in air [71], so they occur in a gaseous medium known as corona discharges. PDs and corona can be initiated when inadequate thickness of the wire insulation allows enough electric field strength in the gap between the wire and a grounded electrode [71], and also due to the presence of water droplets, which aggravate the situation. PDs and corona usually do not generate an appreciable fault current, thus hindering power system protections operation, although they tend to continually damage insulation systems until producing complete failure [71].

PDs and corona usually generate sound, light (mainly UV but also visible), wide frequency range radio interference voltage, electromagnetic wave energy, heat and chemical reactions. Under low pressure environments sound propagates with difficulty, so electromagnetic and UV-visible radiation can be used to detect corona [1,5]. Arcing in short gaps usually is preceded by corona discharges occurring at the region with highest electric field strength [11,78,79]. Corona discharges are key sources of premature failure of insulation systems operating at high altitude [5], occurring earlier at low pressure than at atmospheric conditions [13,80], so surface discharges are among the main causes for early failure of electronic equipment operating in low pressure environments [13]. This is because air density is directly related to the development of electrical discharge development; any reduction of air density leads to a drop of the threshold value of the air ionization electric field, by which the arc is initiated [81]. In [5] it is clearly demonstrated by means of long-exposure photographs that corona inception voltage (CIV) decreases with decreasing values of the atmospheric pressure. Early failure could be initiated because of the fast oxidation due to the ozone generated by the corona discharge [82], although early corona stages may not show any visible effects. Corona effects on insulation materials typically leave a white dust due to the breakdown of the material, but as the condition aggravates, carbon tracks can develop. Other effects include pitting, discoloration as well as micro-crack stains on the insulation. Under the worst case scenario, wires could be severely damaged. It is known that corona inception voltage has an important polarity dependency, since the CIV is lower for negative compared to positive polarity corona, but breakdown for positive polarity occurs at lower voltage than for negative polarity [71,78]. CIV is also influenced by the nature of voltage applied, i.e., DC, AC, switched DC or pulsed among others [5].

Aging effects due to PDs and defects in insulation materials have more impact in AC circuits compared to DC circuits. The likelihood of arc ignition/re-ignition increases with the current and voltage levels, being usually higher in AC circuits. Due to the change of polarity in AC circuits, the arc tends to extinguish with a subsequent re-ignition. The duration of the arc tends to increase under reduced pressure conditions. 

PDs and corona can be detected off- and on-line. Off-line detection methods require the circuit to be de-energized, being usually carried out in special laboratories equipped with voltage sources free of discharges and PD detectors [83]. Off-line detection methods cannot be applied for monitoring PDs and corona activity in real time or in the field, so they are not attractive for an early detection of arc tracking activity in real aircraft environments. On-line detection methods are based on sensors such as high frequency current transformers, i.e., Rogowski coils, transient earth voltage sensors, high frequency sensors including HF/VHF and UHF sensors [84], or acoustic [85] and ultrasonic sensors, which can be used in energized circuits, so some of them can be applied in real time. Corona can also be detected by means of optical spectrophotometers [86], UV sensors [87] or visible-UV cameras [1,78,88,89,90]. However, the disadvantages of some of these methods are their sensitivity to interference from themselves and the surroundings, the difficulties in determining the exact PD-corona location [83], their cost and size. In [12] a simpler method is proposed to detect PDs that is compatible with aeronautic environments since it uses a small low-cost stripped coaxial cable as an antenna sensor, allowing it to reach small places where bigger sensors cannot fit, although it is also susceptible to external interferences. In [1] a low-cost and small-sized sensor that allows location of the corona spots and is compatible with aeronautical environments is applied to detect and locate the corona points in the early stage. In [91] a technique to discriminate between normal and arc fault operation is proposed, which is based on analyzing the magnetic field frequency–domain features using a cost-effective magnetoresistive sensor.

Therefore there is an imperative need to develop cost-effective, small-sized sensors compatible with aircraft environments that are able to locate PD and corona activity in its early stages, before arc tracking effects can develop, thus easing predictive maintenance tasks and safeguarding wiring systems and aircraft integrity.

## 7. Identified Research Needs

This section presents a summary of the research needs identified from the thorough literature review performed in this work, to face the issues and concerns related to arc tracking in insulation systems with special emphasis on aeronautic wire applications.

(1) Arc tracking occurrence depends upon diverse factors such as applied voltage, materials involved and age, environmental conditions (temperature, humidity and pressure), debris type and quantity, and geometry. Changes of such factors will impact the outcome of arc tracking tests, thus making the control of the experiments difficult, and also drawing meaningful conclusions from a reduced number of experimental tests. It is a difficult task to recreate the arc tracking phenomenon under laboratory conditions, since it requires gathering residues and dust in the locations and amounts needed to recreate natural arc tracking conditions, which can be an arduous and non-repeatable task. Generating natural arc tracking conditions at laboratory level is a complex and time-consuming task, often being not cost-effective [40]. Moreover, aeronautic wires can be used under low pressure conditions, typically between 1 bar (sea level conditions) and 0.125 bar (flight altitude in non-pressurized circuits). Specific research and extensive test plans must be carried out to determine wire behavior under arc tracking occurrences in these singular conditions, with a special emphasis on evaluating the transfer of energy from the arc to the material in order to predict the potential damage. Therefore, it is required to develop realistic and standardized arc tracking tests based on extensive test plans to take into account all the abovementioned factors influencing arc tracking occurrence and the effects accounting for aeronautic conditions, specifically reproducing pressure and temperature conditions found in such environments as they have a deep impact on arc tracking occurrence.
(2) As a consequence of point (1), there is a shortage of technical works analyzing in detail the arc tracking phenomenon, and more specifically focused on aeronautic environments. This lack of data is due to the difficulty in characterizing the arc phenomenon due to its irregular behavior [18].
(3) It is known that the shape of the applied voltage waveform (different frequencies, AC, DC, positive and negative polarity, pulsed, impulsive, etc.) has a deep impact on discharge occurrence in insulation systems. There is a lack of studies in this area, and specifically regarding aeronautic environmental conditions. This fact requires a deep research in order to improve the knowledge and to set the baseline for improving or developing new standard test methods accounting for the particularities of aeronautic environments and electrical systems.
(4) Different standards support the design of high voltage systems for aeronautic applications, although standards do not totally cover all high voltage design aspects for aeronautic environments, so there is a growing need in this area in the coming years [71]. For example, the IEC 60664 standard [92] related to insulation coordination for low voltage applications is limited to equipment operating below to 2000 m above sea level, analyzing AC, DC and lightning voltages. It also guides how to calculate creepage distances to avoid tracking based on empirical data, which cannot be applied to equipment operating under low-pressure aeronautic environments [71].
(5) There is the need to develop fast and simple inspection procedures to evaluate wiring system health status. For example, the inclined plane standard test method [74] is commonly used to measure erosion and tracking resistance of insulation materials, since it emulates the thermal degradation by electric arcing under AC supply. However, it is a complex and time-consuming method, being very difficult to perform in field inspection [76], specifically in aeronautic environments. There is also the need to develop an equivalent standard test method for DC supply [75].
(6) Existing AFCB protections actuate when the arc occurs, but not before, so they must be improved to minimize the damage level and shorten the reaction time. There is a need to develop specific electrical protections to detect PD and/or corona activity well before arc tracking occurrence, thus safeguarding electrical wiring systems and aircraft integrity. For this purpose, it is necessary to develop fast response, small-size and cost-effective sensors, as well as specific signal processing techniques specially conceived to operate under aeronautic environmental conditions.
(7) Research on erosion and tracking resistance of insulation materials is a key point to ensure the long-term reliability and safety of electrical insulation systems. For example, such characteristics can be improved by using polymeric nanocomposites. However, studies to analyze the relationship between the role of interfacial strength and the electrical properties of nanocomposites [23] are still scarce, and there are fewer studies analyzing the behavior of such insulation materials under aeronautic environmental conditions.

## 8. Conclusions

This paper has performed a thorough literature review of the current situation of wiring insulation materials for aircraft electrical systems, mainly focused on arc tracking mechanisms, effects, mitigation strategies and research needs. This is a key point in the development of next generation MEAs, since the increase of electrical power and the consequent rise in transmission voltage levels will intensify the electrical stress that wiring insulation systems have to withstand, and the risk of arc occurrence. This situation leads to new challenges and failure modes, with the consequent risks the whole aircraft has to face. This review paper has also underlined different aspects for further improvement in wiring insulation systems, while identifying the research needs required to address the issues and practical impediments that wiring insulation systems for aircraft applications are facing. The needs identified in this work are related to the shortage of experimental data, the difficulty of a realistic reproduction of the arc tracking phenomenon under laboratory conditions due to the complexity of such a phenomenon, the need to adapt and improve current standard test methods for this purpose, the need to develop fast and simple inspection procedures to evaluate wiring system health status, or the need to develop new sensors to detect PD and/or corona activity before arc tracking occurrence to avoid or minimize the damage in insulation wiring systems, as well as the development of suitable signal processing techniques to interpret the data of such sensors.

## Figures and Tables

**Table 1:** Common aircraft wires.

Designation	Insulation Material
M5086/1,2	PVC/Nylon
M81381	Aromatic Polyimide (Kapton®)
M22759/34	Cross-Linked ETFE
M22759/80-92	PTFE/Polyimide/PTFE composite (TKT)
M22759/11	Teflon® (PTFE)
M22759/18	Tezfel® (ETFE)

## Acronyms

AC	Alternating current
AEA	All electric aircraft
AFCB	Arc fault circuit breakers
CIV	Corona inception voltage
CTI	Comparative tracking index
DBD	Dry band discharge
DC	Direct current
ETFE	Ethylene tetrafluoroethylene
FAA	Federal Aviation Administration
Kapton®	Aromatic polyimide
MEA	More electric aircraft
PD	Partial discharge
PI	Polyimide
PVC	Polyvinyl chloride
PTFE (Teflon®)	Polytetrafluoroethylene
SAE	Society of Automotive Engineers
SiR	Silicon rubber
TFE	Tetrafluoroethylene
TKT	Teflon®-Kapton®-Teflon®
UV	Ultraviolet
XL	Cross-link
XLPE	Cross-linked polyethylene
